# Supplementary material for: A fault-intrusion-tolerant system and deadline-aware algorithm for scheduling scientific workflow in the cloud
Source: PeerJ Comput Sci. 2021 Nov 2;7:e747. doi: 10.7717/peerj-cs.747 (PMC8576554; doi:10.7717/peerj-cs.747)
Supplement: Supplemental Information 6 [file peerj-cs-07-747-s006.zip › Myproject1.1/docs/org/workflowsim/failure/FailureMonitor.html]

FailureMonitor


JavaScript is disabled on your browser.


- Overview
- Package
- Class
- Use
- Tree
- Deprecated
- Index
- Help

- Prev Class
- Next Class

- Frames
- No Frames

- All Classes

- Summary:
- Nested |
- Field |
- Constr |
- Method

- Detail:
- Field |
- Constr |
- Method


org.workflowsim.failure

## Class FailureMonitor

- java.lang.Object
- - org.workflowsim.failure.FailureMonitor

- ---

    

  ```
  public class FailureMonitor
  extends java.lang.Object
  ```

  FailureMonitor collects failure information

  Since:
  :   WorkflowSim Toolkit 1.0

- - ### Field Summary

    Fields

    | Modifier and Type | Field and Description |
    | --- | --- |
    | `static java.util.Map` | `index2job` Id to a Job. |
    | `protected static java.util.Map<java.lang.Integer,FailureRecord>` | `jobid2record` JobID to a Failure Record. |
    | `protected static java.util.List<FailureRecord>` | `recordList` All the record list. |
    | `protected static java.util.Map<java.lang.Integer,java.util.ArrayList<FailureRecord>>` | `type2record` Type to a Failure Record. |
    | `protected static java.util.Map<java.lang.Integer,java.util.ArrayList<FailureRecord>>` | `vm2record` VM ID to a Failure Record. |
  - ### Constructor Summary

    Constructors

    Constructor and Description

    `FailureMonitor()`
  - ### Method Summary

    Methods

    | Modifier and Type | Method and Description |
    | --- | --- |
    | `static double` | `analyze(int workflowId, int type)` Update the detected task failure rate based on record lists |
    | `static int` | `getClusteringFactor(FailureRecord record)` Gets the clustering factor |
    | `protected static double` | `getK(double d, double a, double t)` Gets the optimal clustering factor based on analysis |
    | `static void` | `init()` Initialize a FailureMonitor object. |
    | `static void` | `postFailureRecord(FailureRecord record)` A post from a broker so that we can update record list |

    - ### Methods inherited from class java.lang.Object

      `clone, equals, finalize, getClass, hashCode, notify, notifyAll, toString, wait, wait, wait`

- - ### Field Detail


    - #### vm2record

      ```
      protected static java.util.Map<java.lang.Integer,java.util.ArrayList<FailureRecord>> vm2record
      ```

      VM ID to a Failure Record. \*


    - #### type2record

      ```
      protected static java.util.Map<java.lang.Integer,java.util.ArrayList<FailureRecord>> type2record
      ```

      Type to a Failure Record. \*


    - #### jobid2record

      ```
      protected static java.util.Map<java.lang.Integer,FailureRecord> jobid2record
      ```

      JobID to a Failure Record. \*


    - #### recordList

      ```
      protected static java.util.List<FailureRecord> recordList
      ```

      All the record list.


    - #### index2job

      ```
      public static java.util.Map index2job
      ```

      Id to a Job.
  - ### Constructor Detail


    - #### FailureMonitor

      ```
      public FailureMonitor()
      ```
  - ### Method Detail


    - #### init

      ```
      public static void init()
      ```

      Initialize a FailureMonitor object.


    - #### getK

      ```
      protected static double getK(double d,
                double a,
                double t)
      ```

      Gets the optimal clustering factor based on analysis

      Parameters:
      :   `d` - delay
      :   `a` - task failure rate monitored
      :   `t` - task runtime

      Returns:
      :   optimal clustering factor


    - #### getClusteringFactor

      ```
      public static int getClusteringFactor(FailureRecord record)
      ```

      Gets the clustering factor

      Parameters:
      :   `record,` - a request

      Returns:
      :   the clustering factor suggested


    - #### postFailureRecord

      ```
      public static void postFailureRecord(FailureRecord record)
      ```

      A post from a broker so that we can update record list

      Parameters:
      :   `record` - a failure record


    - #### analyze

      ```
      public static double analyze(int workflowId,
                   int type)
      ```

      Update the detected task failure rate based on record lists

      Parameters:
      :   `workflowId,` - doesn't work in this version
      :   `type,` - the type of job or vm

      Returns:
      :   task failure rate


- Overview
- Package
- Class
- Use
- Tree
- Deprecated
- Index
- Help

- Prev Class
- Next Class

- Frames
- No Frames

- All Classes

- Summary:
- Nested |
- Field |
- Constr |
- Method

- Detail:
- Field |
- Constr |
- Method
